# November

**Published:** 1890-11-15

**Authors:** 


### NOVEMBER.

"Autumne, ye barber of ye year," has now almost done its work, and "dreary, dark November" is once more upon us, when "the cheerless empire of the sky to Capricorn, the centaur archer, yields." The 11th of November was held to mark the beginning of winter, or the "Hiemis initium" of the Romans; and on the 13th, the sacred banquet called "Epulium Jovis" took place. There is an ancient sculpture—the fall of Mithias—pictured in Forlong's "Rivers of Life," where Great Taurus, symbol of the sun, is now struck down, pierced through by a Phrygian youth, who had long loved and worshipped him. All the powers of Brumel, the wintry solstice, have combined to emasculate the sun. The storms and winds of winter are keen and hard. The fruits have fallen from the trees, and the torch of life is lowered, while Scipio is trying to destroy the tree itself, as well as the bull. The wintry raven croaks life's dirge with hollow cry. But behold! on the adjacent mountain is hope, and the sign of Isis—the promise of a life yet to come, though many ills must be surmounted ere man again realises spring! As Thompson observes, of November's sun, "faint are his gleams with ineffectual shoots." Nevertheless there are often in November some bright, sunny days, albeit short, and generally terminating in fog and mist and rain. For is not there St. Martin's little summer? November 11th is the feast of St. Martin of Tours, who has given his name, Martinmas, to the farewell gleams of sunshine which occasionally do illuminate the earlier part of the month. As some one, however, observed, "it is many a long century from St. Martin's time, and in the course of ages Nature seems to have grown forgetful of the saint." Last year, however, scepticism was silenced by fine weather, and we may hope for the best. In any case let us remember that November 11th

"Is the day of Martilmasse
When cups of wine should freely pass,
Never heeding winter's fare,
On the day of Martilmasse."

The old Anglo-Saxon name for November was "Blot month," or "Bloody month," probably originating in the custom of slaughtering cattle about Martinmas, for preservation of the meat and consumption during winter, when our ancestors lived pretty much through the winter period on salted flesh. November was the ninth month of the ancient Roumelian year, which began in March; but by the Julian arrangement November became the eleventh month, the year beginning in January. The change, however, has not much altered the character of the season, for an old author tells us that it was always a foggy period in these sea-girt isles; although probably the old author had little conception of a modern London fog. As all Londoners know to their sorrow, it defies bricks and mortar, while cabs, passengers, and even omnibuses, loom ghost-like in the cloud-land which so often envelopes the Metropolis during the months of

November and December. Some one said, of all the depressing things in the world is a genuine London fog. We are not, however, prepared to go thus far. For we think a foggy day in the country may be quite as depressing. When, for instance, "the loosened tempest reigns," and "turns from its depth the discontented deep," while "circling sea-fowl cleave the murky clouds." Let us be just even if we are not generous! A London fog is doubtless distressing to many persons. And it is the excuse for manifold "nips" of spirits, which would not otherwise be taken by those who, with husky voices, excuse themselves under the plea of the fog getting down their throats. But a London fog, on the whole, is not so deleterious to health as the bitter north-east winds of approaching spring, or of some days in autumn when blustering Boreas has less pity than usual for withering leaves. It is on record that there are certain asthmatics who are never so free from their ailment as in London foggy weather. Nevertheless, we cannot well tolerate London fogs for the sake of a few asthmatics, and something certainly ought to be attempted with the view of diminishing the fog demon. It is generally admitted that the rich colour of a London fog is consequent on the atoms of carbon it bears. If we cannot make every householder alter his grate arrangements, at least new buildings might be provided with the required smoke-consuming apparatus. But as it is, we go on adding to our nuisance by allowing the old style of grate in all the numerous new dwellings built in or around the metropolis. Hood called November "the month of megrims," with fog above and fog below. Those who can, endeavour to dispel the megrims by the bright fire, the cup of tea, and the sugar-plums on the mantelshelf, after the receipt of Sidney Smith; or otherwise by the good dinner and the sparkling glass. But the poor are but too often compelled to combat their megrims with hot potatoes or smoking chesnuts, which now appear for sale in the streets. Even at this early period certain signs herald the approach of Christmas. Christmas numbers and engravings appear, and holly and mistletoe may be bought. Charitable people bestir themselves in aid of "misery sore and pierced by wintry winds." And servitors of all kinds become more alert, remembering the near approach of Christmas, and the expectation of gifts.

Of notable days in November we have the 1st, "All Saints" or "All Hallows," when it was the custom to beg bread for the souls of the departed. And November 2nd, "All Souls," when it was the custom to dispense "soul mass-cakes" to the poor. In some parts of the country to "bar out" the master was a sacred rite of schoolboys on November 3rd. The 5th is memorable as the anniversary of the landing of William III. in 1688; also for the "papist conspiracy," for which a special service was appointed. The old saying goes, that "while in this country there's powder and shot, gunpowder treason shall not be forgot." But keeping the 5th of November is nevertheless fast dying away, and in progress of time the guys—if there are any—will come to mean something else. The 9th is Lord Mayor's day. In olden times it was an aquatic procession, led from London Bridge to Lambeth by the barge of the Company of Stationers. When arrived at the palace, they received a present of sixteen bottles of the Archbishop's prime wine, giving in return a copy of several almanacs they had the privilege of printing. Then, as now, the day terminated with feasting, and an old poet asked, "Know ye the land where the juice of the wine, makes aldermen learned and bishops divine?" It was on the 9th of November that a farmers' wife at Uxbridge dreamed John Staines, a bricklayer's server, would become Lord Mayor of London, which eventually came to pass, and reminds us of the story of Whittington. "If you November shooting stars would see, from 12th to 14th watching be." November 22, 1824, is notable as the date of dreadful storms on the English coasts. November 23rd is the festival of St. Clement's, who, the hatters say, discovered felt. Flying from his persecutors, he put wool between his sandals and the soles of his feet, which, it is said, was the origin of felt. Speaking of hats reminds us that hats were first mentioned in history in 1449, when King Charles VII. made his entry into Rouen. People wore hoods previously, and, as with umbrellas, there was great opposition to the introduction of hats. November 29th is the day of "omens." To spill the salt, to cross the knife, to see a winding sheet on the candle, to see a "coffin" jump forth from the fire, to hear a raven croaking, was esteemed more unlucky on the 9th of November than at any other date.

"Or if the babbling fule we call a jay,
A squirrel, or a hare, but cross the way."

Fortunately, although much superstition remains, we are not quite so credulous as our ancestors. We do not now hesitate to start on a journey on a Friday, and a great many do not object to sit down thirteen in number to dinner. But old superstitions, like old religions, take a deal of killing. History demonstrates that wherever ignorance has prevailed, every obscure occurence has been attributed to supernatural agency, and under such influence *inexplicable* and *miraculous* became convertible terms. Are we to thank the School Board and their pianos for the change?

